# PCR diagnostics of *Mycobacterium tuberculosis *in historic human long bone remains from 18^th ^century burials in Kaiserebersdorf, Austria

**DOI:** 10.1186/1756-0500-1-83

**Published:** 2008-09-17

**Authors:** Lutz Bachmann, Barbara Däubl, Charlotte Lindqvist, Luise Kruckenhauser, Maria Teschler-Nicola, Elisabeth Haring

**Affiliations:** 1 Natural History Museum, Department for Zoology, University of Oslo, PO Box 1172, Blindern, NO-0318, Oslo, Norway; 2 Museum of Natural History Vienna, 1st Zoology. Department, Molecular Systematics, Burgring 7, A-1010, Vienna, Austria; 3 Museum of Natural History Vienna, Department of Anthropology, Burgring 7, A-1010, Vienna, Austria; 4 Department of Biological Sciences, The State University of New York at Buffalo, Buffalo, NY 14260, USA

## Abstract

**Background:**

In the present pilot study we applied recently published protocols for detecting *Mycobacterium tuberculosis *in human remains. We screened long bones from an 18^th ^century cemetery and skulls from the anatomical "Weisbach collection" (19^th ^century). In addition, besides the study of abundance of tuberculosis in inmates of the poorhouse itself, we were interested to test whether in this particular instance tuberculosis can be identified from cortical bones, which are rarely affected by tuberculosis, but mostly better preserved than the vertebral bodies or epiphyses.

**Method:**

The DNA extractions from the bone samples were obtained following established ancient DNA protocols. Subsequently extracts were subjected to a series of PCR amplifications using primer pairs published previously [1,2]. PCR products of the expected size were subsequently sequenced.

**Results:**

Only primers targeting the repetitive IS*6110 *insertion sequence yielded PCR products of appropriate size. In one sample only (skull sample WB354 of the "Weisbach collection") sequence analysis revealed an authentic *M. tuberculosis *sequence that matched to a reference sequence from GenBank.

**Conclusion:**

With a variety of established PCR approaches we failed to detect *M. tuberculosis *DNA in historic human femurs from an 18^th ^century cemetery relating to a poor house in Kaiserebersdorf, Austria. Our data may indicate that in this particular case, thoracic or lumbar vertebrae, i.e. bones that are severely affected by the disease, would be more suitable for molecular diagnostics than long bones. However, the unpredictable state of DNA preservation in bones from museum collections does not allow any general recommendation of any type of bone.

## Background

Bone tuberculosis (*Spondylitis tuberculosa*, "Pott's disease") and joint tuberculosis are caused by *Mycobacterium tuberculosis *and appear among others as late manifestations of a tuberculosis infection. Because of the inflammatory bony changes, e.g., partial or total destruction of the vertebral bodies or joint elements, manifestations of tuberculosis infections of (pre)historic human skeletal remains are very often identifiable by means of macroscopic inspections. Since the spread of *M. tuberculosis *depends on population density, its verification is also of high interest for the reconstruction of population dynamic processes in archaeology and anthropology [3]. Nevertheless, post mortem destructions with substantial substance loss or other pathologies with a similar appearance such as, e.g., destruction and remodeling of joint surfaces caused by a fracture and dislocation of a joint element, or an idiopathic avascular necrosis of the femoral head, may lead occasionally to erroneous diagnoses, which may miss- or under-represent the prevalence of tuberculosis in past human populations. An unambiguous molecular identification of tuberculosis for historic human bone remains has therefore been highly appreciated by anthropologists and paleo-epidemiologists.

In recent years, tuberculosis has been diagnosed from a variety of historic human bone remains using ancient DNA methodology. Spigelman and Lemma [4] were the first to detect authentic DNA of *M. tuberculosis *in pre-European-contact human remains from Borneo by means of PCR amplification. This study has been criticized to some extent for technical issues, but the results were confirmed some ten years later [5]. Other examples of detecting *M. tuberculosis *in historic samples include ancient Egyptian mummies [6] and twelve approximately 140 – 1,200 years old mummies excavated from the Andes Mountain region of South America [1]. In seven samples *Mycobacterium *DNA could be detected out of which two proved positive for *M. tuberculosis*. In another study several loci of the *M. tuberculosis *genome were targeted in DNA extracted from naturally mummified remains from three 18^th ^century individuals from Hungary by means of PCR and spoligotyping [7], and *M. tuberculosis *rather than *Mycobacterium bovis *was identified as the cause of the disease. Taylor et al. [2] were able to confirm the morphology based diagnosis of tuberculosis for approximately 2,200 years old human remains excavated from Tarrant Hinton, Dorset, United Kingdom, using a series of sensitive PCR amplifications. In addition, they identified a member of the "modern" type of *M. tuberculosis *and excluded other strains such as *M. bovis *as the source of infection of this particular individual. More recently, *M. tuberculosis *DNA could also be genotyped from five Iron Age individuals from Aymyrlyg, South Siberia [8]. So far, the most comprehensive studies have been conducted by Zink et al. [9,10]. Remains of 41 individuals from the ancient Egyptian population (3,000-500 BC) have been screened for *M. tuberculosis*, among them 21 samples without morphological osseous changes. *M. tuberculosis *was detected in 9 samples including 2 cases without pathological bone changes [9]. In 2005, Zink et al. [10] screened numerous samples of different age and origin, and determined high frequencies of tuberculosis infections in historic populations.

Most studies mentioned above used either dried skin [1] or thoracic and lumbar vertebrae as well as ribs [2,8], i.e. those parts of the skeleton that are affected most by Pott's disease, as sources for DNA extractions. Nevertheless, to some extent long bones have been shown suitable for detection of *M. tuberculosis*, e.g., Fusegawa et al. [11], although vertebrae, spines, and ribs remain the preferred samples. Zink et al. [10] also screened 51 long bones for the presence of *M. tuberculosis*, and showed that such samples may also be suitable for investigating osseous tuberculosis, although with probably lower success rate.

In the present pilot study, we screened 10 long bones from the 18^th ^century burials of the Kaiserebersdorf castle, Austria, for *M. tuberculosis *DNA. In addition to study the abundance of tuberculosis in inmates of the poorhouse itself, we were interested to test whether in this particular instance tuberculosis can be identified from cortical bones (here we used the most robust element, the femur). Such bones are rarely affected by tuberculosis but are frequently much better preserved than the vertebral bodies or epiphyses, and preservation of authentic ancient DNA in the bones' middle regions has been shown likely [12]. According to historical sources, the cemetery was localized close to a chapel and a poor house, which was established by the Habsburg empress Maria Theresia. The skeletal remains of 34 individuals were systematically investigated, and 24 individuals were identified as elder females. Nearly all individuals exhibited severe skeletal changes or pathological alterations. Among them not only progressive degenerative joint lesions indicating long-lasting hard labor, but also features such as cribra orbitalia (anemia), chronic Vitamin C deficiency (newly build bone structures and porosities) and Vitamin D deficiency (rachitic, osteomalacia, osteoporosis) as well as severe inflammations (meningitis and sinusitis) and symptoms of infectious diseases such as syphilis and tuberculosis were observed [13].

## Methods

### Samples

All samples were taken from the collections housed at the Department of Anthropology, Natural History Museum Vienna, Austria. Ten samples were taken from the midshaft femur of ten different individuals recovered from the burial site at the castle of Kaiserebersdorf, south-east of Vienna: KE1, KE8, KE18, KE20, KE22b, KE26b, KE22, KE23, KE26c, KE29. [5]. Tuberculosis was clearly identified in one individual (KE20), a well preserved skeleton of an end-mature female, which shows characteristic osteolytic changes among two vertebral bodies and the adjacent portion of the os sacrum. Individual KE23, a mature female, very likely represents a case of beginning joint tuberculosis. The proximal metaphysis of the left ulna shows an active osteolytic process in form of a cavity. From an anthropological point of view, the inmates of the poor house in Kaiserebersdorf represent a group of hard working, extremely insufficiently nourished people. It is considered likely that infectious diseases such as tuberculosis have spread within this community.

As positive controls, three bone samples (core samples obtained from skull vaults) from the anatomical "Weisbach collection", dated to the end of the 19^th ^century were used. Individual data such as sex, age-of-death, and cause-of-death were recorded in handwritten protocols [14]: WB 565, a 22 year old male, died from meningitis tuberculosa. The internal layer exhibits no signs of inflammation, but many small (1–2 mm in diameter), often confluent depressions, which have been interpreted as features of tubercles. WB 354, a 24 year old male, died from tuberculosis of the peritoneum. The internal layer shows few very tiny new bone formations within the sagittal sinus and small depressions by tubercles in the middle and occipital cranial base. The sample has been taken from a pathomorphologically completely inconspicuous region of the skull. WB 594, a 22 year old male, died from tuberculosis of the peritoneum and meningitis. The internal lamina shows slight newly build bone structures within the sinus and imprints of tubercles exclusively at the posterior cranial base. These skull remains are macroscopically well preserved because they have never been exposed to soil.

Finally, two bone samples (also from the midshaft femur) were taken from the site Hainburg (Lower Austria) that dates back to the early Bronze Age (ca. 2,000-1,500 BC): HB284 (a female, age-of-death 21–24), and HB310 (a female, age-of-death 30–35). From this epoch, bones with morphological alterations due to tuberculosis have never been reported from Austrian localities (N_investigated _= 2000). Therefore, we considered them as negative controls.

### DNA extraction, PCR, and DNA sequencing

Small samples were chopped off the bones and ground into a fine powder using a mortar under liquid nitrogen. Approximately 0.1–0.3 gram of bone powder was subsequently transfered into a 2.0 ml screw-capped centrifuge tube and DNA was extracted according to the silica based protocol with modifications [15,16].

The DNA extracts were subjected to a series of PCR amplifications using primer pairs (table 1) previously described [1,2]. The primer pairs proposed by [1] target the insertion sequence IS*6110*. This insertion was shown to be specific for the *M. tuberculosis *complex [17], and is present in high copy number in most *M. tuberculosis *strains [18]. Targeting the IS*6110 *insertion sequences in the *M. tuberculosis *genome is considered the most sensitive approach [19], and, therefore, most frequently applied. The primer pairs taken from [2] target five different regions of the *M. tuberculosis *genome, including the repetitive IS*1081 *insertion sequences.

**Table 1:** Primers used for amplifying *M. tuberculosis *target DNA

**Primer**	**Length of product**	**Sequence 5' – 3'**	**Reference**
IS1081_F2	135 bp	CTGCTCTCGACGTTCATCGCCG	Insertion sequence IS1081
IS1081_R2		GGCACGGGTGTCGAAATCACG	[2]
IS1081_R3	113 bp	TGGCGGTAGCCGTTGCGC	
OxyR_F3	110 bp	CACTGCCTACGCGACCAGACG	oxidative stress regulator OxyR pseudogene
OxyR _R1		TCTGCGGAATCAGTGTCACC	[2]
OxyR _F4	94 bp	AGACGCTCGATGCTGCCCAA	
pncA_F2	117 bp	GGCGGACTACCATCACGTCG	Pyrazinamidase gene
pncA _R2		GGAGTACCGCTGACGCAATGCGGT	[2]
pncA _R3	96 bp	GGCCACGACGAGGAATAGTC	
D1flanking_F	112 bp	ATCGAAAGGCTAACGGGTGC	flanking sections of TbD1 region
D1flanking _R2		CTGCTCGCCGAGTTGGTCGATA	[2]
D1flanking _R3	96 bp	TCGATACCGTCGATCGTGTCAAAG	
D1internal _F3	115 bp	CGAGCTACGACGCTCGCTATTA	TbD1 region
D1internal _R3		GCATATCGTGATCCGTCTCGAT	[2]
D1internal _R4	105 bp	CTCGATCATCAGTAGTTCGGGATTC	
TbA	TbA/TbB:	CTCGTCCAGCGCCGCTTCGG	insertion sequence IS*6110*
TbB	120 bp	CCTGCGAGCGTAGGCGTCGG	[1]
TbC	TbC/TbD:	GCTTCGGACCACCAGCACCT	
TbD	95 bp	GCGTCGGTGACAAAGGCCAC	

For the primers of [2], F (forward) and R (reverse) denote orientation of the primer and for each gene amplification was performed with the first two primers followed by reamplification with the third primer in combination with one of the original primers.

Target Sequences: IS108 = Insertion sequence IS1081; OxyR = oxidative stress regulator OxyR pseudogene; pncA = Pyrazinamidase gene; D1 = TbD1 region described in [25] that is deleted in modern strains of *M. tuberculosis*; TbA-TbD: insertion sequence IS*6110*.

PCR amplifications were performed using the *PfuTurbo *DNA polymerase (Stratagene, La Jolla, CA, USA) or the DyNAzyme (Finnzymes, Espoo, Finland) DNA polymerase in 1× Reaction Buffer, 0.2 mmol/L of each dNTP, 0.04% bovine serum albumin (BSA), 1 μmol/L of each primer, and 2 μL of DNA extract (DNA concentration not quantified). Thermal cycling conditions were: 94°C for 2 min; 32 cycles of 94°C for 50 sec, 48° – 60°C for 50 sec, and 72°C for 1 min; and a final extension at 72°C for 10 min. PCR products were purified for direct sequencing using 8 μL 10× diluted exoSAP-IT solution (USB Corporation, Cleveland, OH, USA) per reaction. Cycle sequencing, using the same primers as in the PCR reaction, was performed in 10 μL reactions using 2 μL BigDye Terminator Cycle Sequencing Ready Reaction Kit (Applied Biosystems, Foster City, CA, USA), 2 μL 5× Sequencing Buffer, 10 pmol primer, and 3 μL purified PCR product. Sequencing products were purified with ethanol precipitation and analyzed using an ABI 3100 Genetic Analyzer (Applied Biosystems, Foster City, CA, USA).

PCR products that could not be sequenced directly were cloned in the pCR4Blunt-TOPO plasmid vector by means of the TOPO TA Cloning Kit (Invitrogen) and individual clones were subsequently sequenced.

### Inhibitory effect of DNA extracts

Ancient DNA extracts may contain PCR inhibiting components that can cause misleading results. In such instances failure to obtain PCR products cannot be assigned to the absence of the target DNA but simply to inhibition of the reaction. In order to test for co-extracted PCR inhibitors in the samples used in the current study, established test PCRs targeting a ~1000 bp and a ~300 bp fragment of the mitochondrial genome of microtine rodents were spiked with ancient DNA extracts. Spiking PCR amplifications [20] were performed in a final volume of 25 μL using the *Taq *DNA polymerase (Roche) in 1× Reaction Buffer, 0.2 mmol/L of each dNTP, 1 μmol/L of each primer, 50 ng genomic DNA of *Microtus maximowiczii *and 2 μL of DNA extract (DNA concentration not quantified). Thermal cycling conditions were: 94°C for 2 min; 35 cycles of 94°C for 20 sec, 56°/57°C for 30 sec, and 72°C for 60/40 sec; and a final extension at 72°C for 7 min. The primers were *Pro+ *(5'-ACC ATC AGC ACC CAA AGC TG-3') and *Phe*- (5'-AAG CAT TTT CAG TGC TTT GCT T-3') for the ~1000 bp fragment, and *Pro+ *and *mico3*- (5'-GTA AAA GAA GCA TTA ATT AAA-3') for the ~300 bp fragment [21]. None of the ancient DNA extracts inhibited amplification of the test PCRs, as assessed by three replicates. PCR inhibition through ancient DNA extracts was therefore not considered a significant problem for the selected samples.

### Precautions against contamination

The bone samples were processed independently in the DNA labs of the Natural History Museum Vienna and the Natural History Museum Oslo following standards to avoid contamination of samples: These include in particular, usage of DNA, DNAse, and RNAse-free consumables and reagents, as well as pipette tips with aerosol protection filters, and UV-treatment of tubes prior to extraction and PCR amplifications. Negative controls for extractions (without any sample) as well as PCR reactions (without any DNA) were included in the experimental setup. The samples from Hainburg (Lower Austria, Bronze Age) were considered as additional negative controls. Samples from presumably TB-containing cases were never processed before the experiments on the negative controls and samples of unknown infection status had been finished. In both labs no studies of modern mycobacterial or human DNA had been performed before, and especially no modern mycobacterial DNA was used as positive PCR controls.

## Results

When subjecting the long bone samples from Kaiserebersdorf, Austria, as well as the positive and negative controls to PCR amplification targeting various regions in the genome of *Mycobacterium tuberculosis*, several PCR products were obtained. OxyR amplified for the skull WB594 using the primer pair R1/F4. The PCR targeting the IS*6110 *insertion sequences yielded PCR products of appropriate size for the skulls WB354, WB565, and WB594 using primer pair TbA/TbB, and for the long bones KE23 and HB284, and the skulls WB354 and WB594 using primer pair TbC/TbD.

When sequencing the obtained PCR products only WB354 amplified with the primer pair TbC/TbD yielded a sequence identical with the targeted IS*6110 *insertion sequences of *M. tuberculosis *(reference sequence: AF 181860), and was therefore considered positive. The sequence has been deposited in GenBank under the accession number FJ177511. None of the other cloned PCR products yielded sequences with similarity to *M. tuberculosis *but proved unspecific PCR amplification (sequence similarity to, e.g., sequences from *Pseudomonas *or various fungi).

With the exception of the unspecific amplification of non-target DNA of samples KE23 and HB284 when using the TbC/TbD primer pair, DNA extracts from long bones never yielded PCR products that were visible as distinct band of appropriate size on ethidium-bromide stained agarose gels. Even reamplification attempts were negative for the various primer pairs.

## Discussion

In the present study we aimed at diagnosing tuberculosis in historic human long bones from 18^th ^century burials in Kaiserebersdorf, Austria. A positive result, i.e., PCR amplification of a DNA fragment with a nucleotide sequence matching the IS*6110 *insertion sequences of *M. tuberculosis *deposited in GenBank, was only obtained for the skull WB354. This was not unexpected since this sample served as positive control and tuberculosis had been diagnosed earlier in this individual by means of the spoligotyping approach [14]. The amplification of the IS*6110 *insertion sequence proofs the suitability of the applied PCR approach. However, also for this sample positive PCR amplification was only obtained with the primer pair TbC/TbD that targets the repetitive insertion sequences IS*6110*, but not for any of the other molecular markers tested. This may indicate that only very limited amount of template DNA could be extracted from this particular sample. The failure to amplify any tuberculosis specific target DNA from the other positive skull samples may further support this interpretation. It should be considered that a *M. tuberculosis *strain which did not contain IS*6110 *has been reported earlier [22]. Thus, the use of IS*6110 *as a single marker for PCR-facilitated detection *M. tuberculosis *might be insufficient. Nevertheless, the IS*6110 *insertion sequence is very likely the most sensitive marker [19] in the *M. tuberculosis *genome and yielded positive results while other markers such as, e.g., *oxyR *failed [23].

However, even the IS*6110 *insertion sequence could not de determined in the long bones of the samples KE20 and KE23 from the poor house in Kaiserebersdorf, Austria. Given the difficulties in amplifying target DNA of *M. tuberculosis *in the positive skull samples, it may not be surprising that none of the test bones from the burials in Kaiserebersdorf yielded any specific PCR product, although it seems likely from other anthropological evidence that at least samples KE20 and KE23 relate to individuals that suffered from tuberculosis.

Several reasons can account for the negative results on the long bone samples from Kaiserebersdorf: First, some individuals may not have suffered from tuberculosis. This can almost certainly be excluded for samples KE20 and KE23. Second, no DNA was preserved in the samples from Kaiserebersdorf. With the data at hand, we can currently not reject such interpretation. We refrained from amplifying other human target DNA in order to test for the presence of amplifiable DNA. The human actin gene has been suggested for this purpose [24]. However, the samples from Kaiserebersdorf have been handled extensively by humans, and proof of authenticity of any human target DNA would have been close to impossible. Third, the presence of PCR inhibitors in the DNA extracts may have prevented successful amplifications. According to the results of our spiking experiments, this option can be excluded. No sample from Kaiserebersdorf, Austria, prevented successful amplification of high quality test DNA. A further reason for the failure of amplifying *M. tuberculosis *DNA from the samples from Kaiserebersdorf may relate to the bone samples themselves. Long bones such as, e.g., the femurs that are rarely affected by tuberculosis may represent material that is hardly suitable for the molecular detection of *M. tuberculosis*. This interpretation may hold for the particular samples under study, but it has been shown earlier that *M. tuberculosis *can be detected in historic femur samples [10] although with probably lower success rate.

## Conclusion

In the current study, we failed to positively diagnose tuberculosis in DNA extracts from historic human long bones from inmates of a poor house in Kaiserebersdorf, Austria. Using thoracic or lumbar vertebrae for diagnosing tuberculosis, i.e., bones that are severely affected by the disease might have been more successful than long bones. This issue will be further explored in a future study. We conclude that the unpredictable state of DNA preservation in bones from museum collections does not allow any general recommendation of any type of bone.

## Authors' contributions

LB planned the project, conducted the labwork at the NHM, Vienna, and participated in writing and editing the manuscript. BD and LK conducted the labwork at the NHM, Vienna. CL conducted the labwork at the NHM, Oslo. MTN planned the project, contributed the samples, and edited the manuscript. EH planned the project and participated in writing and editing the manuscript. All authors approved the final version of the manuscript.
